# Pilot study on selective antimicrobial effect of a halitosis mouthrinse: monospecies and saliva-derived microbiome in an in vitro model system

**DOI:** 10.1080/20002297.2021.1996755

**Published:** 2021-11-01

**Authors:** Márcia Botelho Dinis, Melissa Agnello, Xuesong He, Wenyuan Shi, Nini Chaichanasakul Tran

**Affiliations:** a School of Dentistry, University of California Los Angeles, Los Angeles, CA, USA; b M2Biome LLC, San Francisco, CA, USA; c The Forsyth Institute, Cambridge, MA, USA

**Keywords:** Antimicrobial, mouthrinse, halitosis, oral microbiome

## Abstract

**Background:**

Halitosis refers to malodor emanating from the oral cavity. Several mouthrinses with halitosis-reduction exist on the market, but their effect on the oral microbiome is largely unknown. In this study, we used an efficient *in vitro* model system to investigate a test mouthrinse's impact on the oral microbiome.

**Methods:**

Single halitosis-associated species and other common oral microorganism cultures were exposed to the test mouthrinse over time, and their viability was determined by culture-based selective plating. Next, the saliva-derived microbiome from healthy and halitosis-associated individuals was cultured in the presence of the test mouthrinse over time using the previously developed *in vitro* model system. The microbiome composition was assessed with *16S rRNA* gene sequencing and downstream bioinformatics analyses.

**Results:**

The test mouthrinse displayed antimicrobial activity against known anaerobic bacterial species producing halitosis-related compounds such as *Fusobacterium nucleatum, F. periodonticum*, and *Prevotella intermedia* but not against other common oral microorganisms. In the multispecies, saliva-derived cultures, mouthrinse exposure decreased the relative abundance of the *Fusobacterium* and *Prevotella* genera while not affecting overall diversity.

**Conclusions:**

The test mouthrinse had promising anti-halitosis characteristics at the microbiome level, as demonstrated by the reduction in the relative abundance of halitosis-associated taxa while maintaining microbial diversity.

## Introduction

Halitosis refers to malodor emanating from the oral cavity [1]. Estimated to affect more than 25% of the population, severe halitosis can be debilitating, resulting in decreased social interactions and overall quality of life [2–4]. Although extra-oral and systemic causes exist, 90% of people suffering from this affliction have halitosis originating in the oral cavity [5]. In addition, oral pathologies such as periodontitis, xerostomia, or mucosal lesions have been associated with malodor [6,7].

The oral cavity consists of complex microbial ecosystems on the teeth, tongue, and saliva. Hundreds of species comprise the oral microbiome, with the vast scope of its functions and contributions to disease recently becoming better understood [8]. Multifactorial in nature, halitosis is caused by the complex microbe–substrate and microbe-microbe interactions and has directly and indirectly been associated with bacteria such as *Fusobacterium periodonticum, Fusobacterium nucleatum, Tannerella forsythia, Prevotella intermedia, Porphyromonas gingivalis*, and *Veillonella atypica* [7,9–15]. Additionally, the production of volatile organic compounds by microbial degradation of proteins, peptides, and amino acids has been implicated as a source of malodor, especially the production of volatile sulfur compounds (VSCs) [16,17]. Various bacterial species present in the oral cavity produce VSCs, and many are also associated with oral conditions such as periodontitis [18,19].

Current halitosis treatments generally focus on reducing the overall bacterial growth through antimicrobial components in mouthrinses. Listerine, a mixture of essential oils originally formulated as a surgical antiseptic, is a commonly used mouthrinse [20]. Other antimicrobial mouthrinses such as chlorhexidine (CHX), cetylpyridinium chloride (CPC), and triclosan are used to control oral pathologies, including halitosis, and have been shown to have a marked effect on oral microbes [21,22]. Recently, Therabreath Fresh Breath Rinse (FBR) with an ‘OXYD-8’, a patented formulation of chlorine dioxide, has been marketed as an anti-halitosis mouthrinse, but its efficacy has not been investigated. We hypothesized that this oxidative component may have an impact on the anaerobic halitosis-associated bacteria. While microbial overgrowth contributes to halitosis, the complexity and highly individualized nature of the oral microbial community creates a challenge in identifying a common cause and effective treatment. To date, the effect of halitosis mouthrinse has mainly been performed on single species, but its impact at the microbial community level has not been investigated due to the lack of a suitable experimental model.

In the current study, we investigated the antimicrobial activity of a test mouthrinse (FBR), specifically marketed to improve halitosis against a panel of individual oral microorganisms. By utilizing our laboratory-developed *in vitro* model system, we demonstrated the effect of the test mouthrinse on saliva-derived oral microbial communities isolated from both healthy volunteers and self-reported halitosis sufferers.

## Materials and methods

### Ethics

The study was approved by the Institutional Review Board at the University of California, Los Angeles (IRB #13-001075 for pooled saliva samples of healthy subjects and 17–001008 for saliva samples of self-reported halitosis subjects). All participants gave informed consent before participating.

### Saliva collection

The healthy saliva community (‘O-mix’) was obtained from pooled saliva samples collected from healthy volunteers. Additionally, saliva was collected from participants with self-reported ‘severe’ halitosis (reported as ‘severely affecting the quality of life’). Participants were recruited and consented via email. Participants were mailed a 50-ml conical tube pre-filled with 75% glycerol with instructions to provide approximately 5 ml of saliva into the tube, seal tightly, and return via express mail to the laboratory. Upon arrival, the saliva samples were aliquoted and stored at −80°C until needed for experiments.

### Mouthrinse antimicrobial activity against single species

Monocultures of strains listed in Table 1 were grown overnight under their respective growth requirements, and subsequently re-inoculated in fresh media, and grown to the exponential phase. At OD_600_ of 0.8–1.0, cultures were spun down, washed twice, and resuspended in phosphate-buffered saline (PBS), the test mouthrinse (FBR, TheraBreath Fresh Breath Rinse, Los Angeles, CA), or Listerine Cool Mint mouthwash (Johnson & Johnson, Skillman, NJ, USA). After 2, 5, 10-, 20-, 30-, and 60-minutes incubation, 1 mL aliquots were collected, spun down, and washed twice with PBS. Serial dilutions and plating on appropriate agar were performed to count colony-forming units (CFU/mL).

**Table 1.** Species and strains and their growth conditions

Species	ATCC number	Medium	Growth conditions
*Fusobacterium periodonticum*	33693	Columbia broth, Columbia blood agar	anaerobic
*Fusobacterium nucleatum*	23726	Columbia broth, Columbia blood agar	anaerobic
*Tannerella forsythia*	43037	SHI medium liquid, agar	anaerobic
*Prevotella intermedia*	49046	TSB supplemented broth, agar	anaerobic
*Veillonella atypica*	17744	BHI+0.06% lactic acid (broth, agar)	anaerobic
*Porphyromonas gingivalis*	33277	Columbia broth, Columbia blood agar	anaerobic
*Streptococcus sanguinis*	10556	BHI broth, agar	microaerophilic
*Streptococcus gordonii*	10558	BHI broth, agar	microaerophilic
*Streptococcus mutans*	UA140	BHI broth, agar	microaerophilic
*Candida albicans*	SN152	TSB broth, agar	aerobic

**Anaerobic**: 0% O_2_, 5% CO_2_, 5% H_2_; **Microaerophilic**: 2.6% O_2,_ 5% CO_2_; **Aerobic**: 21%O_2_, 0.04% CO_2_, 0.9% Ar, 78% N_2_. **BHI**: brain heart infusion; **TSB**: tryptic soy broth; **TSB** supplemented: tryptic soy broth supplemented with yeast extract, hemin, vitamine K, L-cysteine; **SHI**: medium developed in Dr. W Shi’s laboratory [24].

### Multispecies salivary community antimicrobial assay

Pooled saliva from healthy volunteers (‘O-mix’) previously collected [23] and saliva samples from three individuals with self-reported halitosis were thawed from −80°C and cultured overnight at 37°C under microaerophilic conditions (2.6% O_2,_ 5% CO_2_) in SHI media [24]. Cultures were then spun down, washed twice, and resuspended in either PBS, FBR, or Listerine. After 5 minutes and 30 minutes of exposure, aliquots were collected, spun down, washed twice with PBS, resuspended in PBS, and inoculated into 3 ml of fresh SHI media and grown overnight at 37°C under microaerophilic conditions (2.6% O_2,_ 5% CO_2)_, at which time cultures were spun down and immediately frozen until used for DNA extraction.

### DNA extraction and 16S microbiome sequencing

DNA was extracted from pelleted cultures using the Epicenter MasterPure^TM^ DNA purification kit (Lucigen, USA) following the manufacturer’s instructions. Briefly, prior to the kit purification, the pellets were subjected to mechanical digestion with glass beads followed by lysozyme treatment for 2 hours at 37°C [23]. Then, DNA quality and quantity were measured by a UV spectrophotometer (NanoDrop 2000, ThermoFisher Scientific) at 260 nm and 280 nm. Purified DNA was stored at −20°C until use.

Sample preparation for sequencing was performed using a previously published [25]. Preparation of sequencing libraries and sequencing were performed at the UCLA Microbiome Core facility. Briefly, 10–50 ng of DNA was used in a PCR reaction with barcoded V3-V4 primers and purified using AMPure beads (Beckman Coulter). One hundred ng of each library was then pooled, gel-purified, and quantified (Bioanalyzer, Agilent), and 12 PhiXpM of the mixture, spiked with 20% PhiX, was run on a MiSeq (Illumina, San Diego, CA).

### Bioinformatics and statistical analyses

Sequencing reads were de-multiplexed and adaptor sequences removed. Quality filtering removed bad reads and chimeric sequences prior to analysis. Sequencing data were analyzed using QIIME (Quantitative Insights into Microbial Ecology) version 1.9.1 [25]. Sequences were clustered into operational taxonomic units (OTUs) using UCLUST [26], then aligned and taxonomy assigned with the Human Oral Microbiome Database [27] as reference. Alpha diversity (richness) was estimated by calculating the Shannon Diversity Index GraphPad Prism (GraphPad Prism version 8.0.0, GraphPad Software, San Diego, CA), used for visualizations and descriptive statistics.

## Results

### The test mouthrinse (FBR) displays antimicrobial activity against halitosis-associated microbes

**Figure 1.**
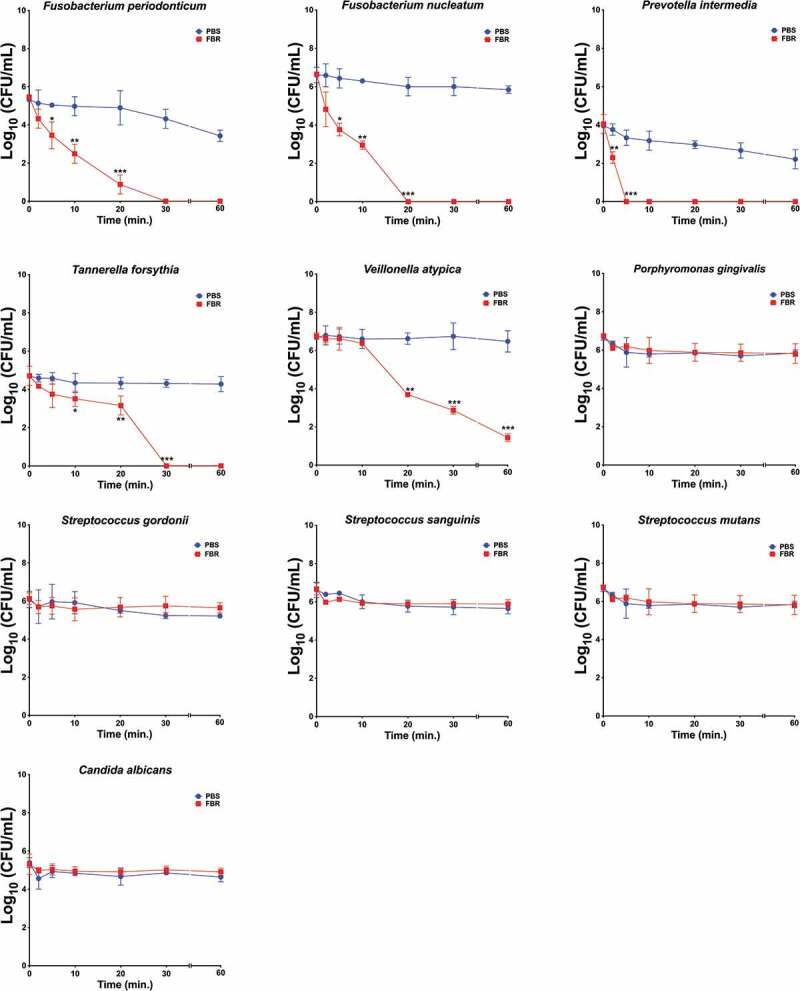
The test mouthrinse antimicrobial activity against common oral species in monoculture. Single-species cultures of *Fusobacterium periodonticum, F. nucleatum, Prevotella intermedia, Tannerella forsythia, Veillonella atypica, Porphyromonas gingivalis, Streptococcus gordonii, S. sanguinis, S. mutans*, and *Candida albicans* were exposed to the test mouthrinse (FBR, red symbol and line) or phosphate-buffered saline solution (PBS, blue symbol and line) for the indicated amount of time, and viability was assessed by CFUs. The curves represent the means and standard deviation of duplicated samples from two independent experiments. Differences in significance between groups were analyzed using two-way anova; *p < 0.05, **p < 0.005, ***p < 0.0005

We first assayed the antimicrobial effect of the test mouthrinse (FBR) on monocultures of a panel of selected oral microbial species (Table 1). Species were selected based on their presumed associations to halitosis or periodontitis, and from other common oral bacteria. Cultures were exposed to FBR or saline solution, and aliquots were collected at each time point and then plated for the CFU quantification (Figure 1, Table 2). FBR exhibited an antimicrobial activity to a subset of the organisms known to be associated with halitosis (Figure 1). After 2 minutes of FBR exposure, the *F. nucleatum* and *F. periodonticum* cultures showed a 95.6% and 82.0% decrease in viability compared to the saline control, respectively. FBR also decreased *T. forsythia* by 86.5% within 5 minutes and displayed a very rapid effect against *P. intermedia* which decreased the CFUs by 96.5% after 2 minutes of exposure. On the other hand, FBR showed a more gradual activity against *V. atypica*, reducing viability by approximately 40% after 10 minutes and reaching 100% killing after 20 minutes. Notably, there was a negligible effect against other common oral microorganisms such as *Streptococcus mutans, P. gingivalis*, or *Candida albicans*, and little-to-no effect on *Streptococcus* species such as *Streptococcus sanguinis* and *Streptococcus gordonii* (Table 2). As a positive control, cultures were also tested in Listerine brand mouthwash, a known effective antimicrobial mouthrinse [20]. In all tested species, treatment with Listerine led to the rapid killing of 100% of the culture in as little as 5 minutes (Table 2).

**Table 2.** Activity of fresh breath rinse against common oral species in monoculture

Associated conditions	Strain	FBR exposure (min)	% Reduction	Listerine exposure (min)	% Reduction
Halitosis and periodontitis [7,9–12]	*Fusobacterium periodonticum*	2	82.0 (± 2.2)	2	22.2 (± 2.0)
		5	97.7 (± 1.1)	5	100.0 (± 0.9)
		10	99.9 (± 0.1)	10	-
		20	100.0 (± 0.4)	20	-
	*Fusobacterium nucleatum*	2	95.6 (± 14.0)	2	13.5 (± 2.0)
		5	99.5 (± 3.3)	5	100.0 (± 1.9)
		10	100.0 (± 0.2)	10	-
		20	-	20	-
	*Tannerella forsythia*	2	61.7 (± 1.0)	2	12.9 (± 1.0)
		5	86.5 (± 7.0)	5	100.0 (± 0.0)
		10	88.0 (± 4.0)	10	-
		20	95.7 (± 1.0)	20	-
Halitosis [9,10,13,14]	*Prevotella intermedia*	2	96.5 (± 3.0)	2	20.2 (± 3.7)
		5	100 (± 2.5)	5	100.0 (± 0.0)
		10	-	10	-
		20	-	20	-
	*Veillonella atypica*	2	24.7 (± 1.9)	2	11.6(± 1.9)
		5	36.5 (± 6.0)	5	100.0 (± 0.0)
		10	40.0 (± 1.0	10	-
		20	99.9 (± 4.3)	20	-
Periodontitis [14,15]	*Porphyromonas gingivalis*	no effect		2	23.7 (± 9.3)
				5	100.0 (± 0.0)
				10	-
				20	-
Commensal [32,33]	*Streptococcus sanguinis*	no effect		2	6.1 (± 2.9)
				5	100.0 (± 0.0)
				10	-
				20	-
	*Streptococcus gordonii*	no effect		2	12.4 (± 4.0)
				5	100.0 (± 0.0)
				10	-
				20	-
Caries [41]	*Streptococcus mutans*	no effect		2	5.2 (± 3.0)
				5	100.0 (± 0.0)
				10	-
				20	-
Stomatitis [42]	*Candida albicans*	no effect		2	12.3 (± 3.9)
				5	100.0 (± 0.0)
				10	-
				20	-

### *The test mouthrinse (FBR) effect on salivary microbial communities using the modified* in vitro *model system*

To test the effects of the mouthrinse on a representative oral microbial community, pooled saliva from healthy volunteers (‘O-mix’) previously collected [23] and saliva collected from three individuals with self-reported ‘severe’ halitosis (referred to as cultures H1, H2, and H3) were cultured separately in a modified *in vitro* model system using SHI media, a specialized medium developed by our laboratory that previously was shown to maintain 80% of the original species diversity [24,28].

Similar to the monoculture experiment, saliva-derived cultures were exposed to either saline, the test mouthrinse (FBR), or Listerine. Aliquots were collected after 5 minutes and 30 minutes of exposure. The community composition of the cultures was assessed at each time point using 16S sequencing. To limit DNA from dead cells interfering with the sequencing results, aliquots were re-cultured in fresh media before DNA was extracted for sequencing. We chose treatment times of 5 minutes and 30 minutes to reflect the practical use of the rinse, with 5 minutes representing the combined time of swishing and gargling suggested on the label and 30 minutes as the time suggested to refrain from eating or drinking after rinsing.

**Figure 2.**
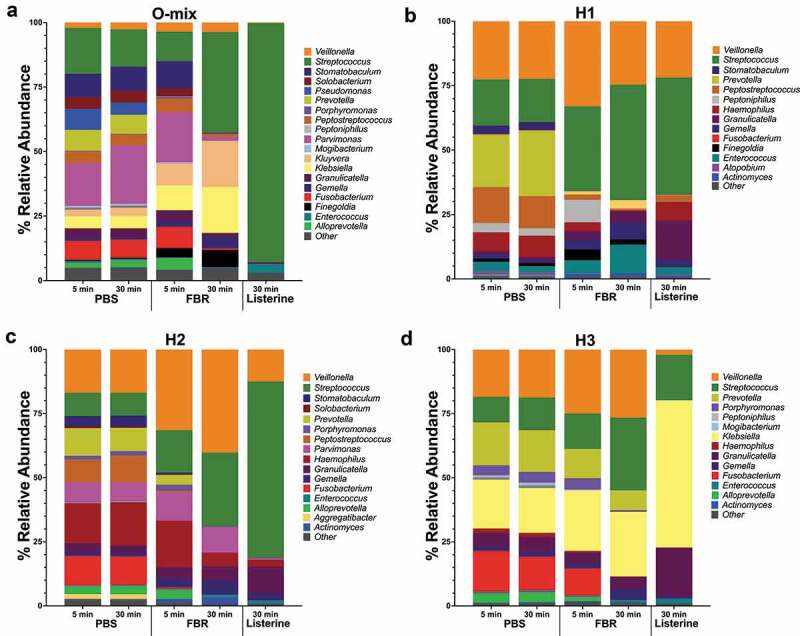
Microbial composition of saliva-derived cultures. Saliva was first cultured in SHI media and subsequently exposed to the test mouthrinse (FBR), phosphate-buffered saline solution (PBS), or Listerine with aliquots collected 5 min and 30 min after treatment. Aliquots were pelleted and then re-cultured in SHI media overnight before extracting DNA and performing 16S sequencing. The relative abundance (percentage) of the genus-level taxonomic results are shown in the stacked bar graphs. (a) O-Mix; pooled saliva from healthy volunteers was used to generate a healthy community culture. (b-d) H1, H2, and H3 refer to saliva-generated cultures with saliva from individuals with self-reported halitosis

**Figure 3.**
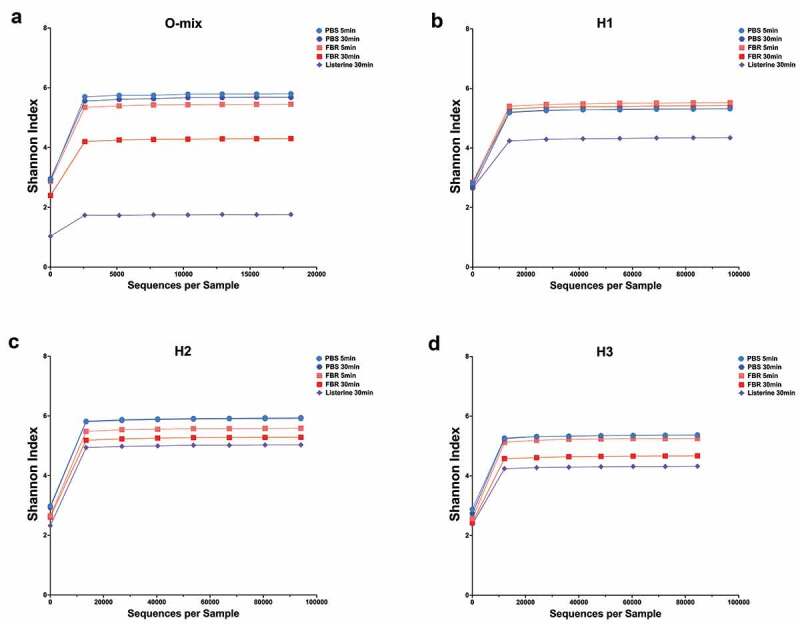
Alpha diversity analysis of saliva-derived microbial communities. The alpha diversity of saliva microbial communities upon treatment with the test mouthrinse (FBR), phosphate-buffered saline solution (PBS), or Listerine (5 min and 30 min after treatment) was analyzed using Shannon index, and the respective rarefaction curves are shown. (a) O-Mix; mixed saliva from healthy volunteers was used to generate a healthy community culture. (b-d) H1, H2, and H3 refer to saliva-generated cultures with saliva from individuals with self-reported halitosis

The relative abundance of identified taxa at the genus level is shown in Figure 2. Visual comparison of the stacked bars revealed a shift in the composition of the FBR-treated samples compared to the saline-treated samples. This marked composition shift was more pronounced after 30 minutes of exposure. There was a striking difference between the saline-treated and FBR-treated samples with less identifiable bacterial taxa at 30-minute exposure time for the Listerine-treated samples. Differences in the relative abundance of the genus-level taxa of interest at each time point are shown in Table 3. The *Fusobacterium* and *Stomatobaculum* genera presented lower abundance in all FBR-treated cultures compared to the saline control. In all halitosis-associated salivary communities, *Prevotella* was less abundant in the FBR-treated cultures compared to the control. Despite taxa-level changes, alpha diversity (Shannon Index) of the communities was relatively unchanged in cultures exposed to FBR after 5 and 30 minutes compared to the control, while the Listerine-treated cultures had much lower richness compared to FBR-treated cultures after 30 minutes (Figure 3).

**Table 3.** Decrease in genus relative abundance after 5 and 30 min exposure to FBR compared to saline control

% Relative abundance after 5 min exposure												
	O-Mix			H1			H2			H3		
	PBS	FBR	↓	PBS	FBR	↓	PBS	FBR	↓	PBS	FBR	**↓**
*Fusobacterium*	7.38	8.27		0.02	0.01	**54.9**	11.51	0.68	**94.1**	15.93	10.69	**32.9**
*Parvimonas*	16.66	19.50		0.01	0.01	**46.5**	8.00	11.01		0.18	0.18	**3.3**
*Peptostreptococcus*	4.45	5.22		13.99	1.97	**85.9**	8.76	0.69	**92.1**	0.01	0.01	
*Porphyromonas*	0.25	0.81		0.00	0.00		1.47	2.38		3.88	4.34	
*Prevotella*	8.10	0.10	**98.7**	20.38	0.24	**98.8**	10.71	3.78	**64.7**	16.74	11.44	**31.7**
*Pseudomonas*	8.18	0.00	**100.0**	0.00	0.00		0.00	0.00		0.00	0.00	
*Solobacterium*	4.53	2.83	**37.5**	0.13	0.00	**98.2**	0.69	0.14	**79.1**	0.05	0.03	**47.6**
*Stomatobaculum*	9.05	10.79		3.33	0.06	**98.2**	4.16	0.81	**80.5**	0.05	0.01	**72.7**
% Relative abundance after 30 min exposure												
	O-Mix			H1			H2			H3		
	PBS	FBR	↓	PBS	FBR	↓	PBS	FBR	↓	PBS	FBR	**↓**
*Fusobacterium*	7.03	0.68	**90.3**	0.02	0.00	**77.8**	11.20	0.01	**99.9**	13.15	0.04	**99.7**
*Parvimonas*	22.71	1.56	**93.1**	0.01	0.01	**9.1**	7.50	9.97		0.21	0.01	**96.8**
*Peptostreptococcus*	4.19	1.11	**73.4**	12.56	0.44	**96.5**	10.53	0.01	**99.9**	0.01	0.01	**43.8**
*Porphyromonas*	0.24	0.05	**79.1**	0.00	0.00		1.68	0.09	**94.8**	4.10	0.68	**83.4**
*Prevotella*	7.40	0.04	**99.5**	25.39	0.05	**99.8**	9.07	0.10	**98.9**	16.30	7.69	**52.8**
*Pseudomonas*	4.71	0.01	**99.9**	0.00	0.00		0.00	0.00		0.00	0.00	
*Solobacterium*	4.53	0.05	**98.9**	0.12	0.00	**98.5**	0.46	0.00	**99.4**	0.00	0.00	
*Stomatobaculum*	9.37	0.31	**96.7**	3.17	0.02	**99.41**	4.36	0.24	**94.6**	0.07	0.00	**97.5**

PBS – phosphate-buffered saline solution; FBR – TheraBreath Fresh Breath Rinse.

↓ – % of decrease.

## Discussion

The human oral cavity harbors one of the most diverse microbiomes in the human body, consisting of bacteria, archaea, protozoa, fungi, and viruses. This complex community plays a crucial role in oral health and disease and systemic health [8,29]. Microbial dysbiosis may underlie many oral health conditions, including halitosis.

Although numerous products and remedies are marketed to consumers claiming to reduce halitosis, most have not undergone rigorous testing [30,31]. In this study, we sought to develop a method to investigate the effects on the oral microbiome of a test mouthrinse (FBR) specifically marketed for halitosis relief. Using a modified *in vitro* model system developed previously in our laboratory [24,28], we performed exposure experiments to monitor how the mouthrinse altered the microbial community. We tested *ex vivo* a pooled ‘healthy’ saliva mix, along with saliva collected from self-reported halitosis sufferers. The results here provide preliminary evidence for more in-depth clinical studies and establish a template for efficient, economical testing for mouthrinses and other oral health care products.

Results from antimicrobial assays against a panel of select oral pathogens, commensals, and known potential contributors to malodor suggest that FBR may specifically target certain halitosis-associated organisms instead of a more generalized antimicrobial activity (Figure 1). The rinse showed activity against halitosis- and periodontitis-associated organisms such as *F. nucleatum* [9,15], *P. intermedia* [10,14], and *V. atypica* [13] but little-to-no activity against oral species typically regarded as commensals, such as *S. sanguinis* and *S. gordonii* (Table 2) [32,33]. In comparison, the Listerine rinse immediately killed all species tested. We used Listerine as a comparison, as it is widely used and has known antimicrobial activity [20].

As halitosis is a complex phenomenon resulting from the production of volatile compounds from various organisms in different niches of the oral cavity [16,34], considering the entire microbial community when testing therapeutics provides more biologically and practically relevant results than single-species assays. Our results with four different saliva-derived cultures (three individual samples from self-reported halitosis sufferers and one sample with pooled saliva from healthy volunteers) overall suggest that the test mouthrinse does not lead to widespread changes and indiscriminate antimicrobial activity. Instead, the diversity and overall structure of the microbial communities were maintained throughout the treatment duration. In comparison, treatment with Listerine resulted in decimating the community structure and the proliferation of a few taxonomic groups.

We observed important changes in abundance at the genus level (Figure 2). The relative abundance of *Fusobacterium* decreased in each culture after 5 minutes of treatment; in one saliva-derived culture from an individual with halitosis, the abundance decreased by 94%. Furthermore, after 30 minutes of exposure, this genus was reduced in all tested saliva communities (Table 3). The *Fusobacterium* genus includes the species *F. nucleatum*, a known producer of volatile sulfur compounds in the oral cavity and previously shown to be increased in abundance in those exhibiting oral malodor [16,17]. Other taxonomic groups of interest for halitosis detected in the cultures include the genus *Porphyromonas*, in which we observed a decrease in abundance in three of the four cultures, and *Prevotella*, which decreased in the cultures derived from halitosis-associated saliva (Table 3). The genus *Porphyromonas* includes *P. gingivalis*, an important periodontal pathogen, and part of the so-called ‘red complex’ of oral pathogens, and a known producer of VSCs [16,35]. Taken together, these results suggest a possible benefit of this test mouthrinse through targeted reduction of halitosis-associated organisms. In the future, the potential clinically- relevant effect of the mouthrinse should be evaluated in a clinical study.

Previous clinical studies have demonstrated a reduction of the volatile sulfur compounds implicated in halitosis through the use of mouthrinses containing antimicrobial ingredients, such as cetylpyridinium chloride [36] and chlorhexidine (CHX). However, there are a limited number of investigations into the overall effects of these products on the oral microbiome. Still, there is some evidence that CHX use leads to a drastic shift in the salivary microbiome [21]. Thus, although a decrease in the total bacterial number may improve halitosis by reducing the overall levels of odorous compounds, it is unclear if this broad approach may lead to detrimental dysbiosis by promoting drastic shifts in the microbial community. Further clinical studies are needed to determine if a targeted approach can lead to more sustainable results.

The test mouthrinse active ingredient is their proprietary ‘OXYD-8’, a patented formulation of chlorine dioxide. The presumed mechanism of action is the creation of an oxygenated local environment that becomes inhospitable to anaerobes. Our observations support that hypothesis, as the rinse showed more potent antimicrobial activity against anaerobes, such as *Fusobacterium* species, while was less effective against organisms known to possess mechanisms to reduce reactive oxygen species (ROS), such as *P. gingivalis* [37], *S. mutans* [38], and *C. albicans* [39].

This study aimed to develop an efficient assay and perform a preliminary investigation into the antimicrobial effects of an oral rinse marketed for bad breath. The modified *in vitro* model system is an efficient first step in generating evidence and establishing the microbiome-focused clinical relevance of a potential therapeutic. In addition to using a mixed saliva sample from healthy volunteers to obtain the oral community cultures, we also recruited individuals with self-reported halitosis as a means of providing additional relevance and generating hypotheses for future clinical testing.

This pilot study was limited by using saliva as the only oral sample type, the self-reported nature of the halitosis phenotype by the individuals in the study, and the inherent limitations of 16S sequencing analysis. The home-based collection method allowed participants to donate saliva samples at home conveniently but limited the collected saliva volume. Nevertheless, comparisons of the microbial composition of saliva with other oral sample types, such as tongue coating, suggest that saliva does indeed contain a sufficient representation of the oral microbiota [40]. We relied on self-reported halitosis experience and cannot be certain of the severity or etiology of each individual’s malodor. Future clinical studies will incorporate halitosis severity and a validated measurement tool to corroborate the individual’s self-report information. Determining the clinical significance of these results will require further investigation in individuals with halitosis.

The results presented here provide evidence that the test mouthrinse has antimicrobial activity against certain halitosis-associated organisms and decreases the abundance of some producers of volatile sulfur compounds within a complex microbial community. Notably, the rinse maintained the microbial diversity of the community while still reducing the abundance of specific taxonomic groups.

## Conclusion

In this study, an efficient *in vitro* assay reflecting the complexity of the oral microbial community was developed to investigate the antimicrobial effects of an oral rinse marketed for halitosis. The test mouthrinse selectively displayed antimicrobial activity against certain halitosis-associated bacteria while maintaining the overall oral microbial community diversity.
